# Taking the knife to neurodegeneration: a review of surgical gene therapy delivery to the CNS

**DOI:** 10.1007/s00701-024-06028-8

**Published:** 2024-03-14

**Authors:** Andrea Perera, Olivier Brock, Aminul Ahmed, Chris Shaw, Keyoumars Ashkan

**Affiliations:** 1 Maurice Wohl Institute of Neuroscience, Department of Basic Clinical Neuroscience, King’s College London, Cutcombe Road, Denmark Hill, London, SE5 9RS UK; 2 Department of Neurosurgery, King’s College Hospital NHS Trust, London, UK; 3 Wolfson Centre for Age-Related Diseases, King’s College London, London, UK; 4 Centre for Brain Research, University of Auckland, 85 Park Road Grafton, Auckland, 1023 New Zealand

**Keywords:** Gene therapy, Regenerative neurosurgery, Neurodegenerative disorders, Viral vector

## Abstract

Gene supplementation and editing for neurodegenerative disorders has emerged in recent years as the understanding of the genetic mechanisms underlying several neurodegenerative disorders increases. The most common medium to deliver genetic material to cells is via viral vectors; and with respect to the central nervous system, adeno-associated viral (AAV) vectors are a popular choice. The most successful example of AAV-based gene therapy for neurodegenerative disorders is Zolgensma© which is a transformative intravenous therapy given to babies with spinal muscular atrophy. However, the field has stalled in achieving safe drug delivery to the central nervous system in adults for which treatments for disorders such as amyotrophic lateral sclerosis are desperately needed. Surgical gene therapy delivery has been proposed as a potential solution to this problem. While the field of the so-called regenerative neurosurgery has yielded pre-clinical optimism, several challenges have emerged. This review seeks to explore the field of regenerative neurosurgery with respect to AAV-based gene therapy for neurodegenerative diseases, its progress so far and the challenges that need to be overcome.

**Supplementary Information:**

The online version contains supplementary material available at 10.1007/s00701-024-06028-8.

## Introduction

The discovery that genetic material could be delivered to cells utilising viral vectors in a safe and efficacious manner has the potential to transform the field of neurodegenerative medicine, offering hope for several previously intractable diseases [26]. In recent years, many genetic mutations have been discovered for diseases such as amyotrophic lateral sclerosis (ALS) that are either directly mechanistic or capable of modifying disease which may be amenable to gene therapy [5, 46]. The major challenge in delivering gene therapy to the central nervous system (CNS) has been achieving suitable tissue biodistribution without off target effects. Although selective serotypes of adeno-associated viral vectors (AAV) can cross the blood-brain barrier (BBB) in infants, even very large doses fail to adequately transduce the brain in older children and adults. Similarly poor results have been observed following intrathecal delivery of AAV vectors [27]. One solution is to circumvent the blood-brain barrier (BBB) by delivering AAV directly to the brain and spinal cord via neurosurgical approaches. Several preclinical studies utilising neurosurgical methods to deliver gene therapies to the brain have recently come to fruition. This review serves to offer an understanding of AAV vectors relevant to neurosurgeons, the caveats to existing delivery approaches and the preclinical basis for neurosurgical gene therapy delivery as well as the challenges faced by clinicians and scientists in this rapidly developing field.

## Viral vectors: an overview

**Fig. 1:**
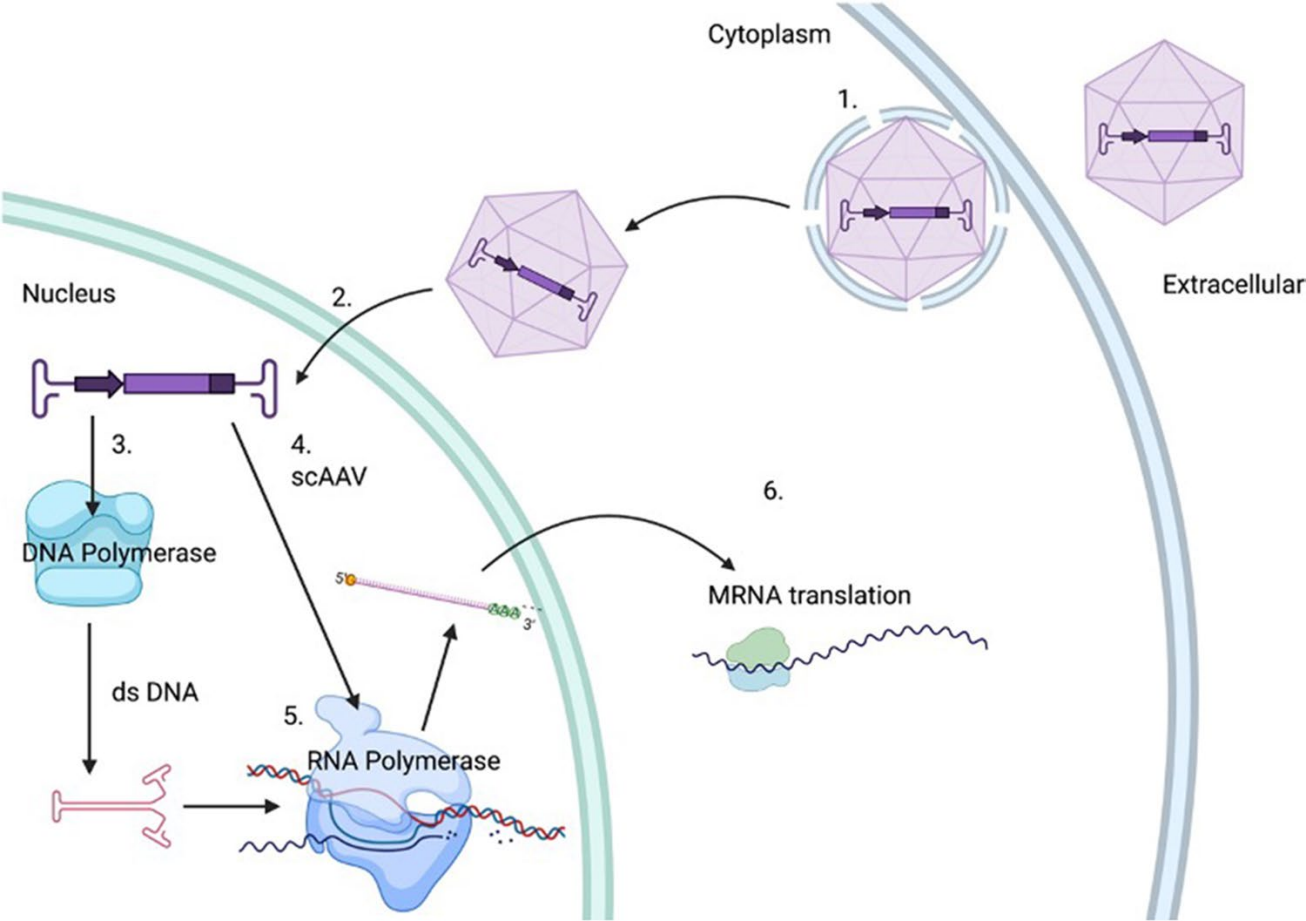
Mechanisms of AAV gene therapy. (1) AAV’s are taken into the cell by endosomes and released into the cytoplasm. (2) The AAV capsid binds to the nuclear membrane, the capsid proteins are then broken down (so the capsid never enters the nucleus) and the vector genome is injected into the nucleus. (3) The single-stranded DNA in the vector genome is then converted to a double-stranded DNA capable of producing mRNA by the cells own DNA polymerase. (4) If the vector genome is self-complementary, then it is already capable of forming the double stranded DNA required and therefore no extra step is required here. (5) RNA polymerase converts the double-stranded DNA to mRNA which is then transported into the cytoplasm and undergoes translation into a protein. It should be noted that the majority of AAV is processed this way and thereby does undergo incorporation into the host genome. A table defining key terms is also shown (figure made with Biorender)

Gene therapy has been defined as a therapy that is ‘used to modify or manipulate the expression of genetic material or to alter the biological properties of a living cell [49]’. Exogenous genetic material must be delivered into the cell via a vehicle and currently the most efficient vehicles are in the form of viral vectors which supplement, silence or edit DNA and RNA (see Fig. 1). There are two main viral vectors which are associated with gene therapy targeting the CNS: adeno-associated viral (AAV) vectors and lentiviral vectors (see Table 1). ProSavin is an example of a lentiviral-based vector targeting Parkinson’s disease, which is delivered directly to the striatum to increase levels of dopamine in transduced cells. Phase 1 studies showed a good safety profile but limited clinical benefit [39]. Despite having a larger packaging capacity, lentiviral vectors have taken a back seat since they integrate into the host genome, which has led to safety concerns regarding insertional mutagenesis and subsequent tumourigenesis [9]. Adeno-associated viral vectors (AAV) have emerged as one of the closest vectors to reaching the clinic in neurodegenerative disease.

**Table 1:** Comparison of lentivirus and adeno-associated virus

Adeno-associated virus	Lentivirus
Advantages	Advantages
• Higher vector titre with high efficiency • Low risk of insertional mutagenesis • Uptake in dividing and non-dividing cells • Different serotypes can provide some tissue preference	• Larger packaging capacity around 10 kb • Integrates into the genome • Uptake in dividing and non-dividing cells
Disadvantages	Disadvantages
• Smaller packaging capacity of 4.8 kb	• Risk of insertional mutagenesis

AAVs are a type of parvovirus belonging to the dependovirus genus, so termed due to its’ inability to replicate without the help of another virus, most commonly adenovirus. AAV is generated by the expression of three genes: ‘rep’ which produces proteins related to replication, ‘cap’ which produces capsid proteins and ‘aap’ which produces assembly proteins [17]. AAV has a capacity of around 4.6 kilobases (kb), while there are efforts to increase this capacity, going beyond this reduces efficiency meaning that some genes cannot effectively fit within the viral capsid restricting its use. The inability of AAV to replicate on its own makes it an attractive choice for gene therapy. There are at least 13 known AAV serotypes (AAV 1–13) [41], approximately 25 nm in size which allows them to easily cross cell membranes and deliver their genetic payload to the cell, leading to long-term transduction [1].

The AAV serotype plays an important role in its biodistribution. AAV’s most associated with the CNS are 1, 2, 5, 8 and 9. Recombinant AAV’s can combine properties of two serotypes such as combining the capsid from a particular serotype and the genome of another such as rAAV2/5 for example utilising the capsid of AAV5 and the genome of AAV2 [19]. Retrograde spread of an AAV is a desirable property as they have a greater biodistribution. AAV 2, 5, 8 and 9 are known to display retrograde transmission [52].

AAVs do not typically integrate within the host genome. The genetic material is packaged between two inverted terminal repeats (ITR), which forms a circular episome once it enters the nucleus and relies on the host cells’ DNA polymerase enzymes to produce the double-stranded DNA required for expression. Alternatively, the DNA can be ‘self-complementary’ which folds to form double-stranded DNA structure without the need for the host DNA polymerase [41] (see Fig. 1). This increases the level and speed of gene expression, however reducing the packaging capacity by half [14].

The type of cell an AAV will transduce depends to some extent on its capsid serotype (termed cellular tropism), but the regulation of transgene expression will depend on its promotor (see Table 2 for key terms). These can be generic promoters commonly derived from cytomegalovirus (CMV) which will express in all cell types or highly specific promoters such as human synapsin (hsyn) promoter, which restricts expression to neurons. This selectivity can be used to reduce toxicity in non-target tissues and off target effects. For example, a vector utilising a *hsyn* promoter will not be expressed in the liver, even if the treatment is delivered systemically. However, the number of cells transduced may be fewer due to the specificity of the vector [6].

**Table 2:** Key terms and definitions

Key word	Definition
Transfection	A method of delivering DNA/RNA into cells without utilising viral vectors
Transduction	Refers to the delivery of genetic material into cells by means of a viral vector
Promoter	A region of DNA upstream of the gene of interest where transcription factors and RNA polymerase can bind—resulting in the transcription of the gene into messenger RNA
Serotype	Serotype refers to the surface antigens displayed by a given viral vector within a known species. AAV serotypes are determined by the sequence of the Cap gene. Serotypes are of importance as they influence cellular tropism and retrograde versus anterograde transport
Retrograde viral transmission	The terminology for retrograde and anterograde neurotransmission largely originates from viral vectors as neuronal tracers. Retrograde virus transmission involves vectors that travel from a location towards the cell body of the neuron (soma)
Anterograde viral transmission	Anterograde virus transmission describes vector travel from the soma of a neuron towards the axonal projections

There are many variables which should be considered when interpreting pre-clinical data relating to translating gene therapies into clinical trials. The above are all examples of important considerations which might affect the biodistribution and efficaciousness of a given therapy outside of the mode of delivery.

## Current approaches to transducing the CNS

**Fig. 2:**
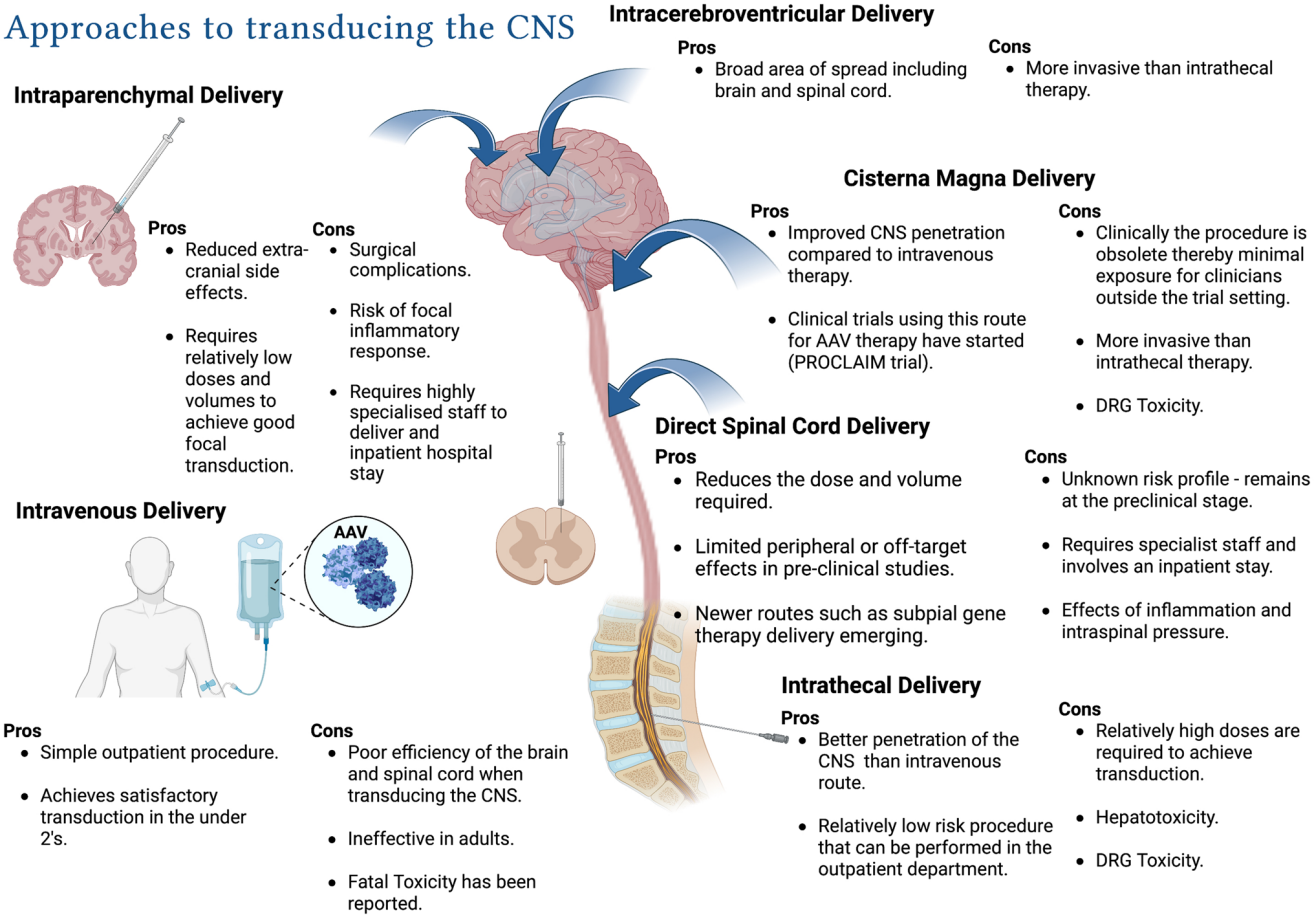
Transducing the CNS. A summary of the different approaches to delivery viral vector-based gene therapy to the central nervous system and the limitations and benefits of each method (figure made with Biorender)

Drug delivery across the blood-brain barrier (BBB) is a universal problem concerning the treatment of many diseases across the CNS (see Fig. 2). There are three existing regulatory approved gene therapies which are all AAV based: Luxturna for congenital Leber’s amaurosis, Zolgensma for spinal muscular atrophy and Glybera an AAV-based gene therapy for lipoprotein lipase deficiency which is no longer available as it was deemed not cost effective to produce [60]. Zolgensma© represents one of gene therapy’s biggest success stories and of the three licensed gene therapies is the only therapy targeting the central nervous system (CNS). Zolgensma is an AAV-9 based, one-time only gene therapy which supplements the survival of motor neuron 1 (SMN1) protein in patients with spinal muscular atrophy (SMA), which is functionally lacking in these patients. They showed remarkable results following a single intravenous weight-based dose in infants under two with 59% achieving functional sitting at 18 months compared with 0% of the control group [13]. This strategy however does not work for adults and older children largely because the doses required are so high, making safe treatment unfeasible. Therefore, another strategy is needed when delivering gene therapy to adults in whom most neurodegenerative conditions manifest.

Pre-clinical work suggested 1/10th of the dose of therapy could be given using lumbar intrathecal delivery compared to an intravenous delivery [57]. Intrathecal delivery of anti-sense oligonucleotide gene therapies has been utilised in several clinical trials in ALS, including in patients with SOD-1 mutations (Toferson) which reduced levels of neurofilament light chains thought to correlate with slowing of disease progression [28, 29]. Anti-sense oligonucleotides (ASOs) are short (typically < 20 bp) strands of nucleotides which can bind mRNA and mark them for degradation. First-generation ASOs were largely ineffective due to degradation and a short half-life. However, newer modifications have meant that they are showing more promise but do require repetitive dosing [45]. Toferson for example is given via a monthly intrathecal infusion. However, the requirement for monthly intrathecal delivery is costly and debilitating for the patient, especially where lumbar puncture is technically challenging. One way to overcome this is the utility of an Ommaya Reservoir to allow percutaneous CSF access. Nusinursen is an ASO utilised in adults with SMA normally given via repeated intrathecal dosing; reports utilising a spinal subdural catheter connected to an Ommaya Reservoir anchored to the lateral abdominal wall can offer an alternative means of administration [40].

A caveat to lumbar intrathecal delivery is the relatively poor biodistribution seen within the spinal cord itself despite relatively high vector doses, especially when considering therapies for diseases such as ALS where spinal cord biodistribution is vital evaluated vector titres of 4.5 × 10^12^ to 1.35 × 10^13^ vector genomes of a recombinant AAV vector with a non-specific chicken beta-actin promoter delivered intrathecally in adult non-human primates [18]. They found that at best 10% of motor neurons in the lumbar spinal cord were transduced, with even lower levels in the cervical and thoracic cord.

Some have argued that better transduction of the brain and cortex of AAV can be achieved via the CSF by intracisterna magna delivery (ICM) in adult non-human primate (NHPs) [18]. ICM showed better biodistribution than lumbar intrathecal delivery with around 20% of motor neurons in the cervical and thoracic cord being transduced and approximately just under 50% of motor neurons being transduced in the lumbar region [17]. When the same group utilised AAV-9 with the same promoter, they saw even lower levels of transduction between 0 and 1.5% in the cervical cord, 8.4–48% in the thoracic cord and 19.3–38.2% in the lumbar cord of adult macaques [18]. ICM as a procedure is now clinically almost obsolete because of periprocedural morbidity prior to the advent of CT guidance. However, with image guidance, the risks can be reduced, and this is being employed for early clinical trials utilising AAV vectors delivered to the cisterna magna. A trial aiming to treat fronto-temporal dementia (FTD) due to *granulin* mutations utilised AAV-9-based progranulin gene replacement delivered via CT-guided ICM delivery in humans began in 2020 with a primary end point of safety (NCT04408625). It should be noted that the study protocol includes triple immunosuppression with methylprednisolone, rituximab and sirolimus to try and mitigate any inflammatory side effects.

Off-target effects of CSF-based gene therapy delivery are noted, namely dorsal root ganglia toxicity which presents as a painful neuropathy and hepatotoxicity [6, 28, 57]. These side effects have placed significant limitations on the utility of CSF-based delivery methods, this coupled with the relatively limited biodistribution have resulted in a search for more efficacious delivery methods.

## DRG toxicity

The dorsal root ganglia (DRG) appear to be vulnerable to toxicity especially via CSF-based routes given their location within the subarachnoid space. A meta-analysis of AAV studies in NHPs showed that DRG pathology started with leucocyte infiltration followed by neuronal loss at intrathecal doses of vector above 1 × 10^12^ vector genomes. No correlation was made between toxicity and capsid serotype, promoter, transgene protein or RNA [21]. A study in NHPs showed that there was no improvement in DRG or sensory axonopathy in animals who had received immunosuppression compared with those who had not, suggesting that the process was not immune mediated [21]. The FDA listed the two major contributors for DRG toxicity as CSF-based administration and high vector doses [16].

## Hepatotoxicity

Hepatotoxicity is a concern in any systemic gene therapy trial as hepatic failure and death in previous gene therapy trials have been reported [22, 23, 42]. However, hepatotoxicity has also been reported following intrathecal delivery in both preclinical and clinical trials. This is one of the strongest rationales for accepting the risk from direct surgical gene therapy in order to reduce the dose and off-target effects. Hepatotoxicity is thought to be driven by T-cell responses and is dose related [15]. Transient increases in liver enzymes can be managed with corticosteroids, but once established can be quickly fatal and the pathophysiology is incompletely understood. All the reported deaths during gene therapy trials from hepatotoxicity have occurred utilising intravenous, systemic administration. The advantage of intraparenchymal CNS delivery is that AAV is almost undetectable in the liver (unpublished observation from Shaw Laboratory).

## Neutralising antibodies and immunosuppression

Exposure to wild-type AAV is very common with up to 60% of the population having previous exposure depending on the serotype and assay used [60]. AAV infection is asymptomatic and therefore transmitted readily amongst humans [47]. Currently many trials exclude patients with high neutralising antibody titres over concerns that the treatment will not be efficacious. One way of overcoming this is to modify the capsid to evade immune detection; however, this can be challenging due to the amount of cross-reactivity between serotypes [60]. A study in non-human primates showed that immunosuppression can reduce the proportion of neutralising antibodies generated to AAV, and immunosuppression did result in systemic side effects however [20]. While it is well established that AAV neutralising antibodies can reduce gene transfer in the systemic circulation, its effect on locally delivered gene therapy to the brain and spinal cord is less well documented. A study where mice underwent intra-thalamic delivery of AAV9 with a luciferase reporter after passive immunisation with seropositive NHP serum showed that only liver transduction was reduced which would be beneficial in preventing off target effects [59].

## Surgical gene therapy

Direct intraparenchymal delivery into the brain emerged as way to reduce the dose of AAV required and thereby alleviate concerns regarding off-target delivery as described above. Several targets were identified, including the nucleus basalis of Meynert to target forebrain neurons involved in Alzheimer’s and the thalamus for cortical transduction [3]. Taking advantage of thalamocortical projections allows the thalamus to be utilised as a target to transduce the cortex which is central to treating neurodegenerative diseases such as fronto-temporal dementia and was validated in NHPs utilising AAV-2 [61]. Many of the trials which have evaluated intraparenchymal gene therapy delivery are based on convection-enhanced delivery.

Convection-enhanced delivery (CED) was pioneered in the 1990s by Dr Edward Oldfield and his team at the National Institute of Health. CED hypothesises that the travel of a compound depends on its free concentration gradient as well as its diffusivity. Diffusivity is inversely proportional to a compound’s molecular weight [7]. CED works on the basis that if a continuous concentration and pressure gradient via slow infusion can be maintained, the biodistribution of the compound of interest is enhanced. Early studies showed that the slower the infusion, the larger the tissue volume that can be reached. This was initially applied to drug for brain tumours; however, it was soon applied to gene therapy delivery [44, 50]. Pre-clinical studies delivering AAV-2-AADC for Parkinson’s disease using convection-enhanced delivery with a rate of 0.1–0.4 µl/min over 60 min were able to transduce cells throughout the striatum despite using smaller volumes [2]. Catheter optimisation for CED can aid biodistribution such as a novel multi-point catheter proposed for combination with CED has been shown to improve biodistribution further in agarose in vitro models [43].

Intraparenchymal delivery of gene therapy to the brain has evolved into phase I/II clinical trials (summary shown in Supplementary Table 1). A clinical trial sponsored by UniQure is using AAV-5 to deliver microRNA targeting mutant huntingtin to the caudate and the putamen with intra-operative MRI and convection-enhanced delivery in patients with Huntington’s disease (NCT04120493). They used a recombinantly engineered AAV which overcomes the obstacle of previous exposure and therefore, in theory, immunity. However, the trial had to be paused in the high-dose cohort temporarily due to safety concerns in three patients who developed symptoms of raised intracranial pressure thought to be due to inflammation. The trial was resumed, with additional safety checks in place; however, no stipulation was given over whether immunosuppression would be mandatory [4]. The trial also utilised a sham surgery arm with skin incisions only. This has several ethical considerations given the nature of the disease and overlooking the impact of sham surgery may have on recruitment, this approach would not control for any form of lesioning effect. Several clinical trials have taken place utilising various stereotaxic intraparenchymal viral vector-based gene therapies, including strategies for Parkinson’s disease and in children for lysosomal storage disorders (Supplementary Table 1) [12].

## Surgical innovation

Neurosurgical gene therapy currently utilises surgical devices which are in their early phases of development. As there are no licensed neurosurgical gene therapy programmes, progress in the surgical technology required to optimise these techniques has been slow and remains relatively niche. Surgical innovation in this space will be imperative towards driving improvements in both efficacy and safety of surgical gene therapy.

The AAV2-AADC intraputaminal delivery trial utilised the Clear Point Neuro-navigation system with the Smart flow© cannulae and markets itself as a cannulae specifically for intraparenchymal drug delivery [11, 12]. This system has the advantage that it allows real-time imaging of the gene therapy infusion allowing biodistribution to be assessed. It should be noted that as with any new technology, a learning curve should be expected. A study reviewing DBS utilising the clear point system navigation system noted higher than expected rates of intracranial haemorrhage, which plateaued as protocols evolved and experience improved [48, 62]. Unfamiliarity with any new technology poses a challenge for gene therapy trials and careful planning and consideration between the surgical team and the sponsors of the technology is required. Recently longer-term convection-enhanced delivery catheters utilising indwelling pumps have become feasible, largely in the field of glioma research with the potential to be applied to gene therapy [51].

### Focussed ultrasound and gene therapy

One pre-clinical approach to improving intraparenchymal delivery without the need for an invasive approach is to utilise focused ultrasound to permeabilise the blood-brain barrier within a specific area in conjunction with peripheral vascular delivery of AAV within microbubbles. Rodent studies showed good biodistribution within the brain regions being targeted, caudate/putamen in this example; however, as this involves reasonably high doses (1.2 × 10^11^vg) delivered into the peripheral vasculature, as expected, the liver, lung and heart were shown to express fluorescence [58]. It is likely that the same concerns and limitations regarding hepatotoxicity that exist for current systemic approaches will apply to this approach. With focussed ultrasound becoming more established in glioma research to permeabilise the blood-brain barrier, it is perceivable that future clinical trials evaluating this technique in gene therapy for neurodegeneration will be on the horizon.

### Enhancing biodistribution and accuracy

Achieving both widespread and specific biodistribution is key to gene therapy delivery. A post-mortem analysis reviewed an unsuccessful surgical gene therapy trial for Alzheimer’s disease which aimed to increase nerve growth factor via AAV-2 by targeting the nucleus basalis of Meynert and utilising its projections to the basal forebrain [10]. However, the neurons they aimed to target did not show transgene expression. They showed that on average the vector travelled less than 1 mm from the catheter tip meaning that the gene therapy itself may have worked but that such a small area was covered that no meaningful therapeutic effect could possibly be expected. The infusion was 20 μl delivered at a rate of 2 μl per min using a non-continuous manual injection at surgery. It was postulated that the failure to utilise convection-enhanced delivery was responsible for the disappointing biodistribution.

In addition to the poor biodistribution, target engagement was absent in several cases due to inaccurate targeting. This trial utilised standard frame-based co-ordinates to target the nucleus basalis of Meynert. Robotic systems are emerging which may improve the accuracy and of stereotaxis, with one simulation showing the (ROSA) robotic system improves accuracy by over 25% compared to traditional frame-based manual techniques [53]. This technology could certainly aid in overcoming this problem for intraparenchymal gene therapy delivery. However, robotic availability as well as the expertise required to utilise this technique will decrease the accessibility of therapies and drive up further the cost of what are already expensive trials. In addition to the utility of robotics, higher resolution and more sophisticated neuroimaging will aid in improving accuracy. Diffuse tensor imaging and tractography sequences can identify white matter tracts with increasing predictability as well as 7 T higher resolution MRI imaging can all serve to improve target identification and planning. A combination of these approaches is likely to maximise chances of successful target engagement in intraparenchymal gene therapy trials.

## Spinal cord

There have been no clinical trials to date delivering AAV vector directly into the spinal cord despite this being the key area of interest for diseases such as ALS. While direct delivery of AAV into the spinal cord has yet to reach clinical trial stage, trials directly delivering stem cells to the spinal cord in patients with ALS and spinal cord injury may aid the development of gene therapies targeting the spinal cord. Surgical delivery of small volumes (15 μl per site) of stem cells into the ventral horn in patients with ALS has been shown to be relatively safe in early trial data [25]. One of the downsides of this approach is that patients required multiple sites of delivery increasing the risks.

An innovative method to overcome this limitation has been proposed from researchers in San Diego where AAV-based gene therapy has been delivered into the subpial space where it spreads along the surface of the spinal cord and diffuses into the parenchyma. Subpial gene therapy delivery allows a larger volume of vector to be delivered safely in mice [54]. Gene silencing of human mutant SOD1 by AAV-9 delivering a short hairpin RNA dramatically reduced human SOD1 mRNA and protein accumulation in transgenic mice and prevented neurological deficits. This model of delivery has been scaled up into pigs and NHPs showing promising results for widespread gene transduction in the spinal cord following a single level subpial infusion [8]. The addition of convection enhanced delivery to this technique may further optimise the spread such that a single level infusion can transduce a large proportion of the spinal cord.

One of the most challenging aspects of direct intraspinal gene therapy delivery is translating the volume, rate and approach used in rodent experiments to larger mammals and ultimately humans. The compliance and tolerance to pressure and the maximal tolerated dose and volume are likely to alter across species in a manner which is not linear to size. The increased availability of intra-operative neuro-monitoring and advances in neuro-imaging techniques will be imperative to improving the safety profile of these delivery techniques.

## Conclusion

As gene therapy, new genetic targets and gene editing technology evolve, more therapies for neurodegenerative diseases are likely to emerge which will depend on adequate delivery to the CNS. The role of neurosurgeons may be key in developing surgical approaches to this complex problem. A multi-disciplinary approach between neurosurgeons, neuroscientists and treating neurologists will hold the key to the ultimate success of this therapy.

## Supplementary Information

Below is the link to the electronic supplementary material.

Supplementary file1 (DOCX 20 KB)

## Data Availability

Not applicable.
