# Supplementary material for: Taking the knife to neurodegeneration: a review of surgical gene therapy delivery to the CNS
Source: Acta Neurochir (Wien). 2024 Mar 14;166(1):136. doi: 10.1007/s00701-024-06028-8 (PMC10940433; doi:10.1007/s00701-024-06028-8)
Supplement: Supplementary file 1 — Supplementary file1 (DOCX 20 KB) [file 701_2024_6028_MOESM1_ESM.docx]

| **Trial Name** | **Intervention and description** | **Status** |
| --- | --- | --- |
| **Safety and Tolerability Study with VY-HTT01 in Adults with Early Manifesting Huntington’s Disease**  **NCT04885114 [30]** | **Gene therapy: rAAV1-miHHT (MicroRNA knock down of HHT)**  Route: Intraparenchymal site not stated  Dose: 4 cohorts from Unilateral low dose delivery of 3.0 x 10^9 vg.ml of rAAV1-miHHT to Bilateral high dose of 9.9x10^10 vg/ml.  Phase: I | Withdrawn (0 Participants recruited)  Reason given by Voyager Therapeutics that this was due to the company pursuing less invasive methods by optimising proprietary capsid design. |
| **Intracerebral Gene Therapy in Children with Sanfilippo Type B Syndrome**  **NCT03300453 [56]** | **Gene therapy: rAAV2/5 hNAGLU (Human Alpha-N-acetylglucosaminidase cDNA)**  Route: Intracerebral administration. Administration over 16 sites to occur simultaneously. 12 Supratentorial white matter, and 4 cerebellar deposits.  Dose: 960uL 4 x 10^12 vg total.. Each deposit was 60uL containing 2.4x10^11 total vg.  Delivered simultaneously at a rate of 0.5uL/minute over 2 hours using coaxial silica glass injection capillaries.  Included immunosuppressive therapy. (Oral Tacrolimus for 2 weeks before surgery and mycophenolate mofetil) Prednisolone was given to patients 1 day before surgery and continued for 10 days after.  Phase: I/II | Completed.  4 patients were included.  6 severe adverse events were reported including raised Alanine aminotransferase (1), diarrhoea during follow up (2) respiratory tract infection (3). No direct adverse events were noted relating to the surgical procedure itself.  Improvement was seen in the four patients injected in terms of cognitive outcomes. |
| **A Dose-escalation and safety and efficacy study of AXO-AAV-GM2 in Tay-Sachs or Sandhoff Disease**  **NCT04669535 [31]** | **Gene therapy: AXO-AAVrh.8-HEXA/B cDNA**  Route: Bilateral Intrathalamic and dual ICM/Intrathecal administration  Dose: Starting, low, medium and high dose vector genomes not stated.  Phase: I | Recruiting  First patient reported to have been dosed in 2021. |
| **Safety and Proof-of-Concept (POC) Study with AMT-130 in adults with early manifest Huntington’s disease.**  **NCT05243017 [32]** | **Gene therapy: rAAV5-miHTT (microRNA)**  Route: Intrastriatal versus sham (skin incisions only no burr holes)  Dose: Low dose 6 x 10^12 vg/subject, High dose 6 x 10^13 vg/subject  Phase: I/II | Recruiting |
| **A Study to evaluate AB-1001 striatal administration in adults with early manifest Huntington’s Disease**  **NCT05541627 [33]** | **Gene therapy: AAVrh10.CAG.hCYP46A1 (human cholesterol 24 hydroxylase gene)**  Route: Bilateral Intrastriatal  Dose: Low 4 x 10^8 vg/uL and High dose 1.1 x 10^9 vg/uL. | Recruiting complete, study ongoing |
| **Safety Study of AADC gene therapy (VY-AADC01) for Parkinson’s Disease (AADC)**  **NCT01973543 [12]** | **Gene therapy: AAV2-hAADC**  Route: MRI guided convection enhanced delivery into the Putamen  Dose: Dose 1: 7.5 x 10^11 vg, Dose 2: 1.5 x 10^12 vg, Dose 3: 4.7 x 10^12 vg, Dose 4: 4.7 x 10^12.  Phase I | Completed  No serious adverse events were noted in the initial phase 1b trial. |
| **Long Term Safety and Efficacy Study of ProSavin in Parkinson’s Disease**  **NCT01856439 [38]** | **Gene therapy: ProSavin (Lentivirus expressing aromatic amino acid dopa decarboxylase, tyrosine hydroxylase and GTP-cyclohydrolase 1).**  Route: Bilateral Intrastriatal  Dose: Low Dose: 1.9 x 10^7 TU, Mid Dose 4.0 x 10^7 TU and High Dose 1x10^8 TU.  Phase: I/II | Completed: 15 patients enrolled  No serious adverse events from surgery or gene therapy. 54 Adverse events recorded of which 3 were moderate. Most common adverse event was on-medication dyskinesias. |
| **Lentiviral Gene Therapy for X-ALD**  **NCT03727555 [34]** | **Gene therapy: Lentivirus expressing TYF-ABCD1**  Route: Intracerebral Lentiviral delivery  Dose: Not stated  Phase: I/II | Completed  No results published yet. |
| **Intracerebral gene therapy for children with early onset forms of metachromatic leukodystrophy (TG-MLD)**  **NC T01801709 [35]** | **Gene therapy: AAVrh.10 ARSA cDNA**  Route: 12 injections; 6 image-guided white matter tracts, with 2 deposits per tract.  Dose: Low Dose 1 x 10^12 vg total, High dose 4 x 10^12 vg total.  Phase I/II | Recruiting complete, study ongoing |
| **A phase I/II trial for treatment of Aromatic L-amino-acid dexarboxylase (AADC) Deficiency using AAV2-hAADC (AADC)**  **NCT01395641 [55]** | **Gene therapy:AAV2-hAADC**  Route: Bilateral Putamen Infusions  Dose: 1.81 x 10^11^vg  Phase I/II | Completed  26 patients in total underwent the treatment.  14 post-surgical adverse events were noted; most notable was transient hypotension in 6 patients, followed by CSF leakage (n=3). |
| **Lentiviral Gene Therapy for MLD**  **NCT03725670 [36]** | **Gene therapy: Lentivirus expressing TYF-ARSA**  **Route: Intracerebral injection**  **Dose: Unknown**  **Phase I/II** | Unknown status |
| **Intracerebral gene therapy for Sanfilippo type A syndrome**  **NCT2053064, NCT01474343 [37]** | **Gene therapy: AAV10-SGSH/AAV10-SUMF1 (SAF-301)**  **Route 6 image guided tracts with 2 deposits per tract.**  **Dose: 7.2 x 10^11^ vg [23]** | Completed  SAF-302 is currently in phase 2/3 trials |

Supplemental Table 1| Clinical trials that have utilised surgical delivery of either lentivirus or adeno-associated viral delivery of gene therapy. The NCBI clinical trials database was searched for the terms ‘intracerebral’ or ‘intraparenchymal’ ‘gene therapy’.
